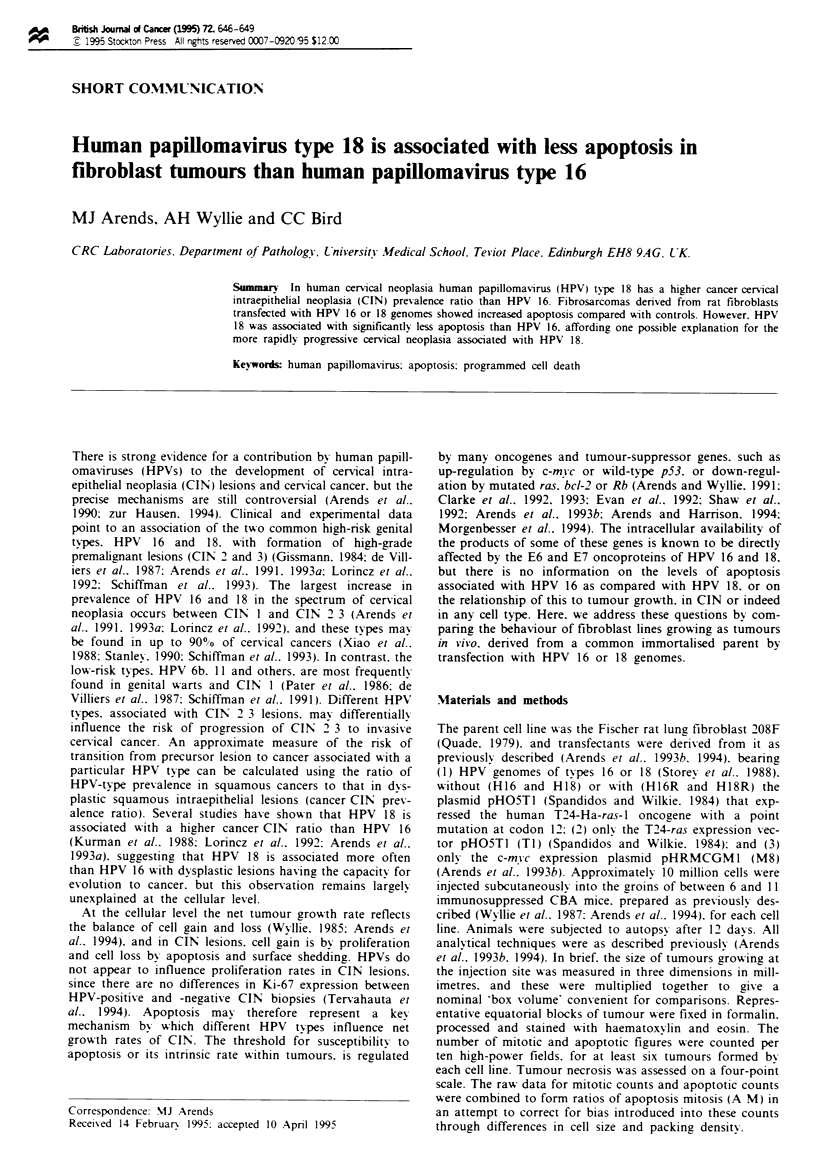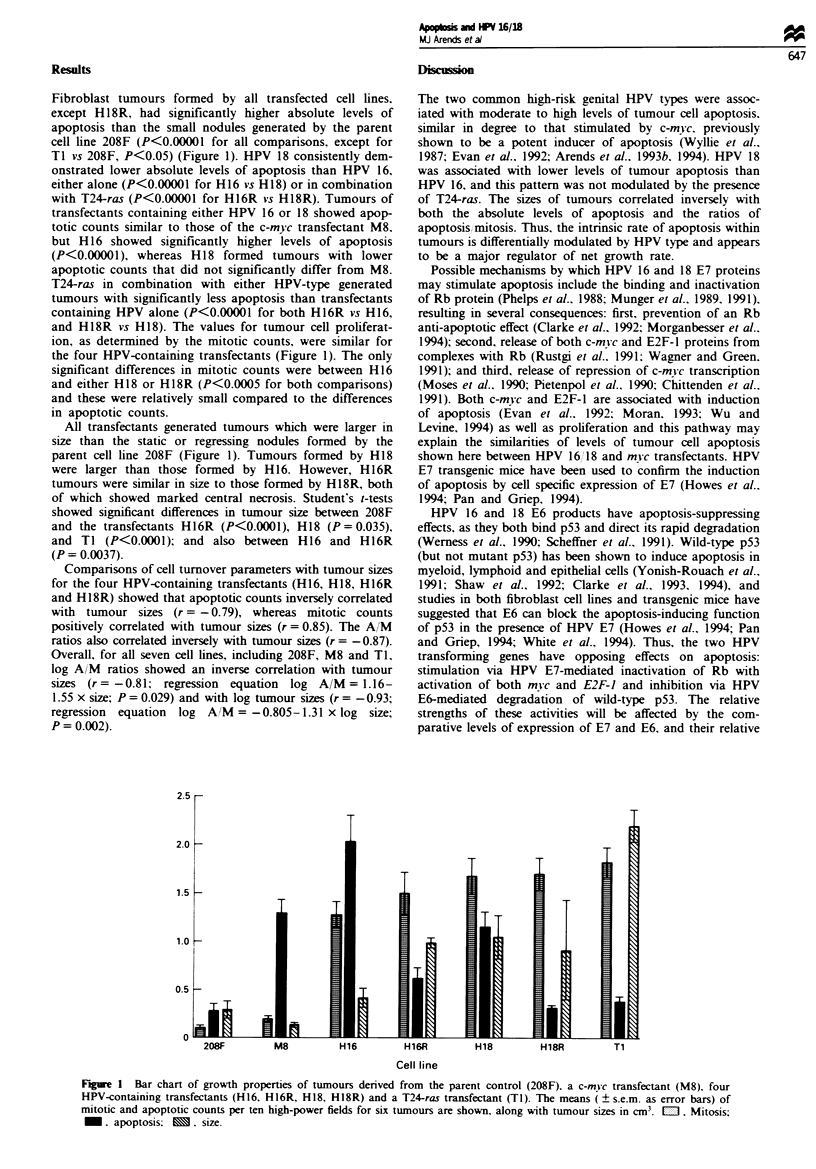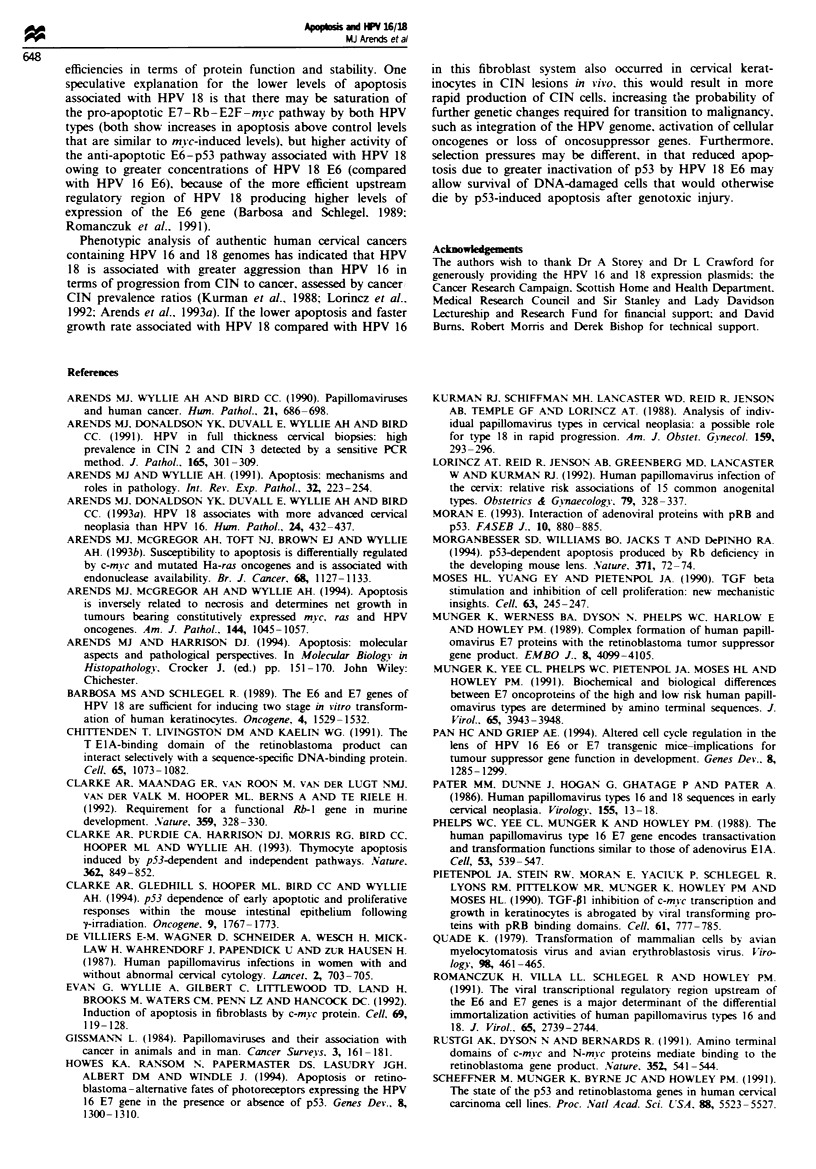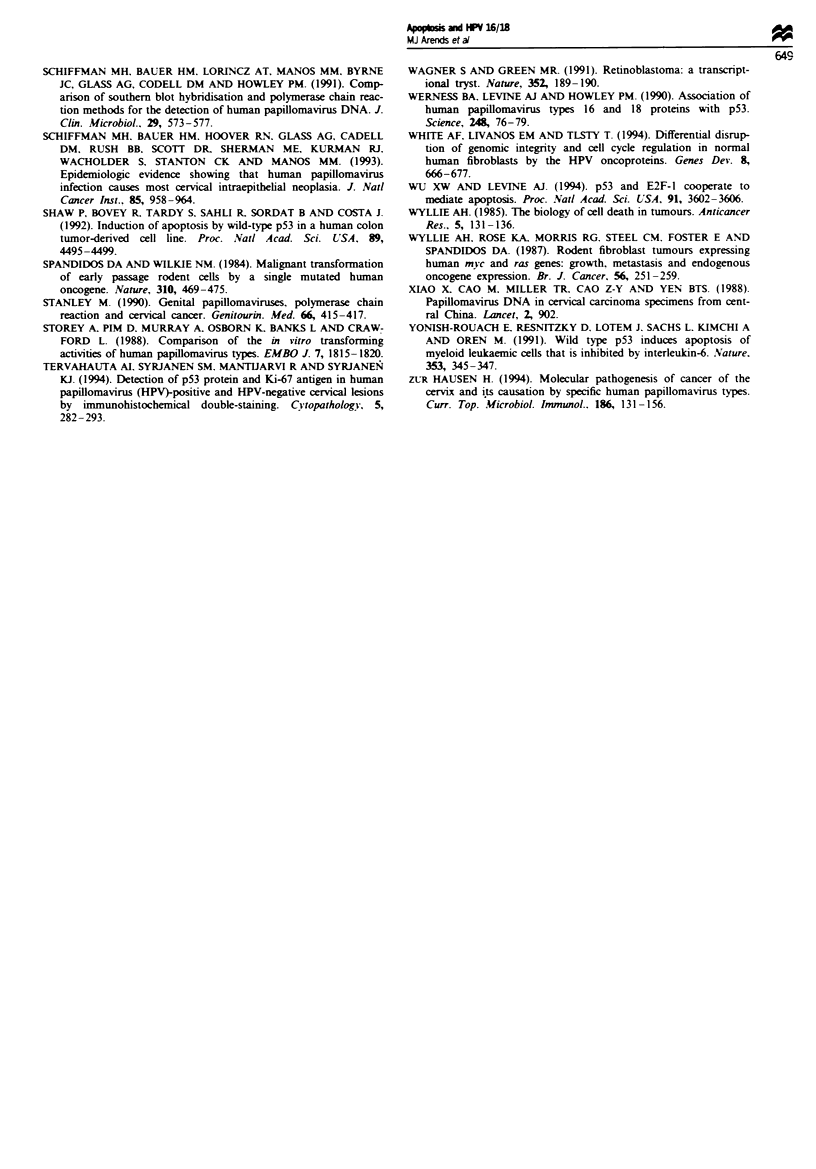# Human papillomavirus type 18 is associated with less apoptosis in fibroblast tumours than human papillomavirus type 16.

**DOI:** 10.1038/bjc.1995.388

**Published:** 1995-09

**Authors:** M. J. Arends, A. H. Wyllie, C. C. Bird

**Affiliations:** Department of Pathology, University Medical School, Edinburgh, UK.

## Abstract

In human cervical neoplasia human papillomavirus (HPV) type 18 has a higher cancer/cervical intraepithelial neoplasia (CIN) prevalence ratio than HPV 16. Fibrosarcomas derived from rat fibroblasts transfected with HPV 16 or 18 genomes showed increased apoptosis compared with controls. However, HPV 18 was associated with significantly less apoptosis than HPV 16, affording one possible explanation for the more rapidly progressive cervical neoplasia associated with HPV 18.


British Journal of Cancer (1995) 72, 646-649

© 1995 Stockton Press All rights reserved 0007-0920/95 $12.00

SHORT COMMUNICATION

### Human papillomavirus type 18 is associated with less apoptosis in fibroblast tumours than human papillomavirus type 16

MJ Arends, AH Wyllie and CC Bird

*CRC Laboratories, Department of Pathology, University Medical School, Teviot Place, Edinburgh EH8 9AG, UK.*

**Summary** In human cervical neoplasia human papillomavirus (HPV) type 18 has a higher cancer cervical intraepithelial neoplasia (CIN) prevalence ratio than HPV 16. Fibrosarcomas derived from rat fibroblasts transfected with HPV 16 or 18 genomes showed increased apoptosis compared with controls. However, HPV 18 was associated with significantly less apoptosis than HPV 16, affording one possible explanation for the more rapidly progressive cervical neoplasia associated with HPV 18.

**Keywords:** human papillomavirus; apoptosis; programmed cell death

There is strong evidence for a contribution by human papillomaviruses (HPVs) to the development of cervical intraepithelial neoplasia (CIN) lesions and cervical cancer, but the precise mechanisms are still controversial (Arends *et al.*, 1990; zur Hausen, 1994). Clinical and experimental data point to an association of the two common high-risk genital types, HPV 16 and 18, with formation of high-grade premalignant lesions (CIN 2 and 3) (Gissmann, 1984; de Villiers *et al.*, 1987; Arends *et al.*, 1991, 1993*a*; Lorincz *et al.*, 1992; Schiffman *et al.*, 1993). The largest increase in prevalence of HPV 16 and 18 in the spectrum of cervical neoplasia occurs between CIN 1 and CIN 2/3 (Arends *et al.*, 1991, 1993*a*; Lorincz *et al.*, 1992), and these types may be found in up to 90% of cervical cancers (Xiao *et al.*, 1988; Stanley, 1990; Schiffman *et al.*, 1993). In contrast, the low-risk types, HPV 6b, 11 and others, are most frequently found in genital warts and CIN 1 (Pater *et al.*, 1986; de Villiers *et al.*, 1987; Schiffman *et al.*, 1991). Different HPV types, associated with CIN 2/3 lesions, may differentially influence the risk of progression of CIN 2/3 to invasive cervical cancer. An approximate measure of the risk of transition from precursor lesion to cancer associated with a particular HPV type can be calculated using the ratio of HPV-type prevalence in squamous cancers to that in dysplastic squamous intraepithelial lesions (cancer/CIN prevalence ratio). Several studies have shown that HPV 18 is associated with a higher cancer/CIN ratio than HPV 16 (Kurman *et al.*, 1988; Lorincz *et al.*, 1992; Arends *et al.*, 1993*a*), suggesting that HPV 18 is associated more often than HPV 16 with dysplastic lesions having the capacity for evolution to cancer, but this observation remains largely unexplained at the cellular level.

At the cellular level the net tumour growth rate reflects the balance of cell gain and loss (Wyllie, 1985; Arends *et al.*, 1994), and in CIN lesions, cell gain is by proliferation and cell loss by apoptosis and surface shedding. HPVs do not appear to influence proliferation rates in CIN lesions, since there are no differences in Ki-67 expression between HPV-positive and -negative CIN biopsies (Tervahauta *et al.*, 1994). Apoptosis may therefore represent a key mechanism by which different HPV types influence net growth rates of CIN. The threshold for susceptibility to apoptosis or its intrinsic rate within tumours, is regulated by many oncogenes and tumour-suppressor genes, such as up-regulation by c-*myc* or wild-type *p53*, or down-regulation by mutated *ras*, *bcl-2* or *Rb* (Arends and Wyllie, 1991; Clarke *et al.*, 1992, 1993; Evan *et al.*, 1992; Shaw *et al.*, 1992; Arends *et al.*, 1993*b*; Arends and Harrison, 1994; Morgenbesser *et al.*, 1994). The intracellular availability of the products of some of these genes is known to be directly affected by the E6 and E7 oncoproteins of HPV 16 and 18, but there is no information on the levels of apoptosis associated with HPV 16 as compared with HPV 18, or on the relationship of this to tumour growth, in CIN or indeed in any cell type. Here, we address these questions by comparing the behaviour of fibroblast lines growing as tumours *in vivo*, derived from a common immortalised parent by transfection with HPV 16 or 18 genomes.

### Materials and methods

The parent cell line was the Fischer rat lung fibroblast 208F (Quade, 1979), and transfectants were derived from it as previously described (Arends *et al.*, 1993*b*, 1994), bearing (1) HPV genomes of types 16 or 18 (Storey *et al.*, 1988), without (H16 and H18) or with (H16R and H18R) the plasmid pHO5T1 (Spandidos and Wilkie, 1984) that expressed the human T24-Ha-*ras*-1 oncogene with a point mutation at codon 12; (2) only the T24-*ras* expression vector pHO5T1 (T1) (Spandidos and Wilkie, 1984); and (3) only the c-*myc* expression plasmid pHRMCGM1 (M8) (Arends *et al.*, 1993*b*). Approximately 10 million cells were injected subcutaneously into the groins of between 6 and 11 immunosuppressed CBA mice, prepared as previously described (Wyllie *et al.*, 1987; Arends *et al.*, 1994), for each cell line. Animals were subjected to autopsy after 12 days. All analytical techniques were as described previously (Arends *et al.*, 1993*b*, 1994). In brief, the size of tumours growing at the injection site was measured in three dimensions in millimetres, and these were multiplied together to give a nominal 'box volume' convenient for comparisons. Representative equatorial blocks of tumour were fixed in formalin, processed and stained with haematoxylin and eosin. The number of mitotic and apoptotic figures were counted per ten high-power fields, for at least six tumours formed by each cell line. Tumour necrosis was assessed on a four-point scale. The raw data for mitotic counts and apoptotic counts were combined to form ratios of apoptosis/mitosis (A/M) in an attempt to correct for bias introduced into these counts through differences in cell size and packing density.

Correspondence: MJ Arends
Received 14 February 1995; accepted 10 April 1995

### Results

Fibroblast tumours formed by all transfected cell lines, except H18R, had significantly higher absolute levels of apoptosis than the small nodules generated by the parent cell line 208F ($P<0.00001$ for all comparisons, except for T1 *vs* 208F, $P<0.05$) (Figure 1). HPV 18 consistently demonstrated lower absolute levels of apoptosis than HPV 16, either alone ($P<0.00001$ for H16 *vs* H18) or in combination with T24-*ras* ($P<0.00001$ for H16R *vs* H18R). Tumours of transfectants containing either HPV 16 or 18 showed apoptotic counts similar to those of the c-*myc* transfectant M8, but H16 showed significantly higher levels of apoptosis ($P<0.00001$), whereas H18 formed tumours with lower apoptotic counts that did not significantly differ from M8. T24-*ras* in combination with either HPV-type generated tumours with significantly less apoptosis than transfectants containing HPV alone ($P<0.00001$ for both H16R *vs* H16, and H18R *vs* H18). The values for tumour cell proliferation, as determined by the mitotic counts, were similar for the four HPV-containing transfectants (Figure 1). The only significant differences in mitotic counts were between H16 and either H18 or H18R ($P<0.0005$ for both comparisons) and these were relatively small compared to the differences in apoptotic counts.

All transfectants generated tumours which were larger in size than the static or regressing nodules formed by the parent cell line 208F (Figure 1). Tumours formed by H18 were larger than those formed by H16. However, H16R tumours were similar in size to those formed by H18R, both of which showed marked central necrosis. Student's *t*-tests showed significant differences in tumour size between 208F and the transfectants H16R ($P<0.0001$), H18 ($P=0.035$), and T1 ($P<0.0001$); and also between H16 and H16R ($P=0.0037$).

Comparisons of cell turnover parameters with tumour sizes for the four HPV-containing transfectants (H16, H18, H16R and H18R) showed that apoptotic counts inversely correlated with tumour sizes ($r=-0.79$), whereas mitotic counts positively correlated with tumour sizes ($r=0.85$). The A/M ratios also correlated inversely with tumour sizes ($r=-0.87$). Overall, for all seven cell lines, including 208F, M8 and T1, log A/M ratios showed an inverse correlation with tumour sizes ($r=-0.81$; regression equation log $A/M=1.16-1.55\times$ size; $P=0.029$) and with log tumour sizes ($r=-0.93$; regression equation log $A/M=-0.805-1.31\times$ log size; $P=0.002$).

### Discussion

The two common high-risk genital HPV types were associated with moderate to high levels of tumour cell apoptosis, similar in degree to that stimulated by c-*myc*, previously shown to be a potent inducer of apoptosis (Wyllie *et al*., 1987; Evan *et al*., 1992; Arends *et al*., 1993*b*, 1994). HPV 18 was associated with lower levels of tumour apoptosis than HPV 16, and this pattern was not modulated by the presence of T24-*ras*. The sizes of tumours correlated inversely with both the absolute levels of apoptosis and the ratios of apoptosis/mitosis. Thus, the intrinsic rate of apoptosis within tumours is differentially modulated by HPV type and appears to be a major regulator of net growth rate.

Possible mechanisms by which HPV 16 and 18 E7 proteins may stimulate apoptosis include the binding and inactivation of Rb protein (Phelps *et al*., 1988; Munger *et al*., 1989, 1991), resulting in several consequences: first, prevention of an Rb anti-apoptotic effect (Clarke *et al*., 1992; Morganbesser *et al*., 1994); second, release of both c-*myc* and E2F-1 proteins from complexes with Rb (Rustgi *et al*., 1991; Wagner and Green, 1991); and third, release of repression of c-*myc* transcription (Moses *et al*., 1990; Pietenpol *et al*., 1990; Chittenden *et al*., 1991). Both c-*myc* and E2F-1 are associated with induction of apoptosis (Evan *et al*., 1992; Moran, 1993; Wu and Levine, 1994) as well as proliferation and this pathway may explain the similarities of levels of tumour cell apoptosis shown here between HPV 16/18 and *myc* transfectants. HPV E7 transgenic mice have been used to confirm the induction of apoptosis by cell specific expression of E7 (Howes *et al*., 1994; Pan and Griep, 1994).

HPV 16 and 18 E6 products have apoptosis-suppressing effects, as they both bind p53 and direct its rapid degradation (Werness *et al*., 1990; Scheffner *et al*., 1991). Wild-type p53 (but not mutant p53) has been shown to induce apoptosis in myeloid, lymphoid and epithelial cells (Yonish-Rouach *et al*., 1991; Shaw *et al*., 1992; Clarke *et al*., 1993, 1994), and studies in both fibroblast cell lines and transgenic mice have suggested that E6 can block the apoptosis-inducing function of p53 in the presence of HPV E7 (Howes *et al*., 1994; Pan and Griep, 1994; White *et al*., 1994). Thus, the two HPV transforming genes have opposing effects on apoptosis: stimulation via HPV E7-mediated inactivation of Rb with activation of both *myc* and *E2F-1* and inhibition via HPV E6-mediated degradation of wild-type p53. The relative strengths of these activities will be affected by the comparative levels of expression of E7 and E6, and their relative

**Figure 1** Bar chart of growth properties of tumours derived from the parent control (208F), a c-*myc* transfectant (M8), four HPV-containing transfectants (H16, H16R, H18, H18R) and a T24-*ras* transfectant (T1). The means ($\pm$ s.e.m. as error bars) of mitotic and apoptotic counts per ten high-power fields for six tumours are shown, along with tumour sizes in $cm^3$. ▤, Mitosis; ■, apoptosis; ▨, size.

efficiencies in terms of protein function and stability. One speculative explanation for the lower levels of apoptosis associated with HPV 18 is that there may be saturation of the pro-apoptotic E7-Rb-E2F-*myc* pathway by both HPV types (both show increases in apoptosis above control levels that are similar to *myc*-induced levels), but higher activity of the anti-apoptotic E6-p53 pathway associated with HPV 18 owing to greater concentrations of HPV 18 E6 (compared with HPV 16 E6), because of the more efficient upstream regulatory region of HPV 18 producing higher levels of expression of the E6 gene (Barbosa and Schlegel, 1989; Romanczuk *et al*., 1991).

Phenotypic analysis of authentic human cervical cancers containing HPV 16 and 18 genomes has indicated that HPV 18 is associated with greater aggression than HPV 16 in terms of progression from CIN to cancer, assessed by cancer/CIN prevalence ratios (Kurman *et al*., 1988; Lorincz *et al*., 1992; Arends *et al*., 1993*a*). If the lower apoptosis and faster growth rate associated with HPV 18 compared with HPV 16 in this fibroblast system also occurred in cervical keratinocytes in CIN lesions *in vivo*, this would result in more rapid production of CIN cells, increasing the probability of further genetic changes required for transition to malignancy, such as integration of the HPV genome, activation of cellular oncogenes or loss of oncosuppressor genes. Furthermore, selection pressures may be different, in that reduced apoptosis due to greater inactivation of p53 by HPV 18 E6 may allow survival of DNA-damaged cells that would otherwise die by p53-induced apoptosis after genotoxic injury.

**Acknowledgements**
The authors wish to thank Dr A Storey and Dr L Crawford for generously providing the HPV 16 and 18 expression plasmids; the Cancer Research Campaign, Scottish Home and Health Department, Medical Research Council and Sir Stanley and Lady Davidson Lectureship and Research Fund for financial support; and David Burns, Robert Morris and Derek Bishop for technical support.

**References**

ARENDS MJ, WYLLIE AH AND BIRD CC. (1990). Papillomaviruses and human cancer. *Hum. Pathol.*, **21**, 686-698.
ARENDS MJ, DONALDSON YK, DUVALL E, WYLLIE AH AND BIRD CC. (1991). HPV in full thickness cervical biopsies: high prevalence in CIN 2 and CIN 3 detected by a sensitive PCR method. *J. Pathol.*, **165**, 301-309.
ARENDS MJ AND WYLLIE AH. (1991). Apoptosis: mechanisms and roles in pathology. *Int. Rev. Exp. Pathol.*, **32**, 223-254.
ARENDS MJ, DONALDSON YK, DUVALL E, WYLLIE AH AND BIRD CC. (1993*a*). HPV 18 associates with more advanced cervical neoplasia than HPV 16. *Hum. Pathol.*, **24**, 432-437.
ARENDS MJ, McGREGOR AH, TOFT NJ, BROWN EJ AND WYLLIE AH. (1993*b*). Susceptibility to apoptosis is differentially regulated by c-*myc* and mutated Ha-*ras* oncogenes and is associated with endonuclease availability. *Br. J. Cancer*, **68**, 1127-1133.
ARENDS MJ, McGREGOR AH AND WYLLIE AH. (1994). Apoptosis is inversely related to necrosis and determines net growth in tumours bearing constitutively expressed *myc*, *ras* and HPV oncogenes. *Am. J. Pathol.*, **144**, 1045-1057.
ARENDS MJ AND HARRISON DJ. (1994). Apoptosis: molecular aspects and pathological perspectives. In *Molecular Biology in Histopathology*, Crocker J. (ed.) pp. 151-170. John Wiley: Chichester.
BARBOSA MS AND SCHLEGEL R. (1989). The E6 and E7 genes of HPV 18 are sufficient for inducing two stage *in vitro* transformation of human keratinocytes. *Oncogene*, **4**, 1529-1532.
CHITTENDEN T, LIVINGSTON DM AND KAELIN WG. (1991). The T E1A-binding domain of the retinoblastoma product can interact selectively with a sequence-specific DNA-binding protein. *Cell*, **65**, 1073-1082.
CLARKE AR, MAANDAG ER, VAN ROON M, VAN DER LUGT NMJ, VAN DER VALK M, HOOPER ML, BERNS A AND TE RIELE H. (1992). Requirement for a functional *Rb*-1 gene in murine development. *Nature*, **359**, 328-330.
CLARKE AR, PURDIE CA, HARRISON DJ, MORRIS RG, BIRD CC, HOOPER ML AND WYLLIE AH. (1993). Thymocyte apoptosis induced by *p53*-dependent and independent pathways. *Nature*, **362**, 849-852.
CLARKE AR, GLEDHILL S, HOOPER ML, BIRD CC AND WYLLIE AH. (1994). *p53* dependence of early apoptotic and proliferative responses within the mouse intestinal epithelium following γ-irradiation. *Oncogene*, **9**, 1767-1773.
DE VILLIERS E-M, WAGNER D, SCHNEIDER A, WESCH H, MICKLAW H, WAHRENDORF J, PAPENDICK U AND ZUR HAUSEN H. (1987). Human papillomavirus infections in women with and without abnormal cervical cytology. *Lancet*, **2**, 703-705.
EVAN G, WYLLIE A, GILBERT C, LITTLEWOOD TD, LAND H, BROOKS M, WATERS CM, PENN LZ AND HANCOCK DC. (1992). Induction of apoptosis in fibroblasts by c-*myc* protein. *Cell*, **69**, 119-128.
GISSMANN L. (1984). Papillomaviruses and their association with cancer in animals and in man. *Cancer Surveys*, **3**, 161-181.
HOWES KA, RANSOM N, PAPERMASTER DS, LASUDRY JGH, ALBERT DM AND WINDLE J. (1994). Apoptosis or retinoblastoma-alternative fates of photoreceptors expressing the HPV 16 E7 gene in the presence or absence of p53. *Genes Dev.*, **8**, 1300-1310.
KURMAN RJ, SCHIFFMAN MH, LANCASTER WD, REID R, JENSON AB, TEMPLE GF AND LORINCZ AT. (1988). Analysis of individual papillomavirus types in cervical neoplasia: a possible role for type 18 in rapid progression. *Am. J. Obstet. Gynecol.* **159**, 293-296.
LORINCZ AT, REID R, JENSON AB, GREENBERG MD, LANCASTER W AND KURMAN RJ. (1992). Human papillomavirus infection of the cervix: relative risk associations of 15 common anogenital types. *Obstetrics & Gynaecology*, **79**, 328-337.
MORAN E. (1993). Interaction of adenoviral proteins with pRB and p53. *FASEB J.*, **10**, 880-885.
MORGANBESSER SD, WILLIAMS BO, JACKS T AND DePINHO RA. (1994). p53-dependent apoptosis produced by Rb deficiency in the developing mouse lens. *Nature*, **371**, 72-74.
MOSES HL, YUANG EY AND PIETENPOL JA. (1990). TGF beta stimulation and inhibition of cell proliferation: new mechanistic insights. *Cell*, **63**, 245-247.
MUNGER K, WERNESS BA, DYSON N, PHELPS WC, HARLOW E AND HOWLEY PM. (1989). Complex formation of human papillomavirus E7 proteins with the retinoblastoma tumor suppressor gene product. *EMBO J.*, **8**, 4099-4105.
MUNGER K, YEE CL, PHELPS WC, PIETENPOL JA, MOSES HL AND HOWLEY PM. (1991). Biochemical and biological differences between E7 oncoproteins of the high and low risk human papillomavirus types are determined by amino terminal sequences. *J. Virol.*, **65**, 3943-3948.
PAN HC AND GRIEP AE. (1994). Altered cell cycle regulation in the lens of HPV 16 E6 or E7 transgenic mice-implications for tumour suppressor gene function in development. *Genes Dev.*, **8**, 1285-1299.
PATER MM, DUNNE J, HOGAN G, GHATAGE P AND PATER A. (1986). Human papillomavirus types 16 and 18 sequences in early cervical neoplasia. *Virology*, **155**, 13-18.
PHELPS WC, YEE CL, MUNGER K AND HOWLEY PM. (1988). The human papillomavirus type 16 E7 gene encodes transactivation and transformation functions similar to those of adenovirus E1A. *Cell*, **53**, 539-547.
PIETENPOL JA, STEIN RW, MORAN E, YACIUK P, SCHLEGEL R, LYONS RM, PITTELKOW MR, MUNGER K, HOWLEY PM AND MOSES HL. (1990). TGF-β1 inhibition of c-*myc* transcription and growth in keratinocytes is abrogated by viral transforming proteins with pRB binding domains. *Cell*, **61**, 777-785.
QUADE K. (1979). Transformation of mammalian cells by avian myelocytomatosis virus and avian erythroblastosis virus. *Virology*, **98**, 461-465.
ROMANCZUK H, VILLA LL, SCHLEGEL R AND HOWLEY PM. (1991). The viral transcriptional regulatory region upstream of the E6 and E7 genes is a major determinant of the differential immortalization activities of human papillomavirus types 16 and 18. *J. Virol.*, **65**, 2739-2744.
RUSTGI AK, DYSON N AND BERNARDS R. (1991). Amino terminal domains of c-*myc* and N-*myc* proteins mediate binding to the retinoblastoma gene product. *Nature*, **352**, 541-544.
SCHEFFNER M, MUNGER K, BYRNE JC AND HOWLEY PM. (1991). The state of the p53 and retinoblastoma genes in human cervical carcinoma cell lines. *Proc. Natl Acad. Sci. USA*, **88**, 5523-5527.

SCHIFFMAN MH, BAUER HM, LORINCZ AT, MANOS MM, BYRNE JC, GLASS AG, CODELL DM AND HOWLEY PM. (1991). Comparison of southern blot hybridisation and polymerase chain reaction methods for the detection of human papillomavirus DNA. *J. Clin. Microbiol.*, **29**, 573–577.

SCHIFFMAN MH, BAUER HM, HOOVER RN, GLASS AG, CADELL DM, RUSH BB, SCOTT DR, SHERMAN ME, KURMAN RJ, WACHOLDER S, STANTON CK AND MANOS MM. (1993). Epidemiologic evidence showing that human papillomavirus infection causes most cervical intraepithelial neoplasia. *J. Natl Cancer Inst.*, **85**, 958–964.

SHAW P, BOVEY R, TARDY S, SAHLI R, SORDAT B AND COSTA J. (1992). Induction of apoptosis by wild-type p53 in a human colon tumor-derived cell line. *Proc. Natl Acad. Sci. USA*, **89**, 4495–4499.

SPANDIDOS DA AND WILKIE NM. (1984). Malignant transformation of early passage rodent cells by a single mutated human oncogene. *Nature*, **310**, 469–475.

STANLEY M. (1990). Genital papillomaviruses, polymerase chain reaction and cervical cancer. *Genitourin. Med.* **66**, 415–417.

STOREY A, PIM D, MURRAY A, OSBORN K, BANKS L AND CRAWFORD L. (1988). Comparison of the *in vitro* transforming activities of human papillomavirus types. *EMBO J.* **7**, 1815–1820.

TERVAHAUTA AI, SYRJANEN SM, MANTIJARVI R AND SYRJANEN KJ. (1994). Detection of p53 protein and Ki-67 antigen in human papillomavirus (HPV)-positive and HPV-negative cervical lesions by immunohistochemical double-staining. *Cytopathology*, **5**, 282–293.

WAGNER S AND GREEN MR. (1991). Retinoblastoma: a transcriptional tryst. *Nature*, **352**, 189–190.

WERNESS BA, LEVINE AJ AND HOWLEY PM. (1990). Association of human papillomavirus types 16 and 18 proteins with p53. *Science*, **248**, 76–79.

WHITE AF, LIVANOS EM AND TLSTY T. (1994). Differential disruption of genomic integrity and cell cycle regulation in normal human fibroblasts by the HPV oncoproteins. *Genes Dev.* **8**, 666–677.

WU XW AND LEVINE AJ. (1994). p53 and E2F-1 cooperate to mediate apoptosis. *Proc. Natl Acad. Sci. USA*, **91**, 3602–3606.

WYLLIE AH. (1985). The biology of cell death in tumours. *Anticancer Res.*, **5**, 131–136.

WYLLIE AH, ROSE KA, MORRIS RG, STEEL CM, FOSTER E AND SPANDIDOS DA. (1987). Rodent fibroblast tumours expressing human *myc* and *ras* genes: growth, metastasis and endogenous oncogene expression. *Br. J. Cancer*, **56**, 251–259.

XIAO X, CAO M, MILLER TR, CAO Z-Y AND YEN BTS. (1988). Papillomavirus DNA in cervical carcinoma specimens from central China. *Lancet*, **2**, 902.

YONISH-ROUACH E, RESNITZKY D, LOTEM J, SACHS L, KIMCHI A AND OREN M. (1991). Wild type p53 induces apoptosis of myeloid leukaemic cells that is inhibited by interleukin-6. *Nature*, **353**, 345–347.

ZUR HAUSEN H. (1994). Molecular pathogenesis of cancer of the cervix and its causation by specific human papillomavirus types. *Curr. Top. Microbiol. Immunol.*, **186**, 131–156.